# Editorial: Minimal residual disease (MRD) assessment in multiple myeloma patients

**DOI:** 10.3389/fonc.2023.1211935

**Published:** 2023-05-22

**Authors:** Angelo Maiolino, Elaine Sobral Da Costa, Alberto Orfao

**Affiliations:** ^1^Department of Internal Medicine, Universidade Federal do Rio de Janeiro, Rio de Janeiro, Brazil; ^2^Department of Hematology, Instituto Americas de Ensino, Pesquisa e Inovação, Rio de Janeiro, Brazil; ^3^Translational and Clinical Research Program, Cancer Research Center (IBMCC, USAL-CSIC), Cytometry Service (NUCLEUS) Institute for Biomedical Research of Salamanca (IBASAL), CIBERONC and Department of Medicine, University of Salamanca, Salamanca, Spain

**Keywords:** minimal residual disease, multiple myeloma, prognostic factor, disease monitoring, flow cytometry

Multiple myeloma (MM) is a neoplastic plasma cell disorder characterized by the expansion and accumulation of clonal plasma cells. The introduction of novel drugs and new therapeutic options has led to significantly higher complete response rates and prolonged progression-free and overall survival (1). Despite these advances, MM remains an incurable disease. Thus, there is a need to refine response criteria and methods for more sensitive identification of persistence of lower levels of minimal (measurable) residual disease (MRD) (2). The International Myeloma Working Group has defined the response criteria for patients with MM by including MRD (3). Moreover, depth response based on MRD has emerged as one of the most important independent prognostic factors in MM and has been tested as a dynamic tool for treatment/disease monitoring, prognostication, and as a new (potential) therapeutic endpoint in clinical trials and drug approval for MM patients (4).

The so-called Next Generation Flow (NGF) approach developed by the EuroFlow Consortium (www.euroflo.org) is a high sensitive method to evaluate MRD by flow cytometry, which has been validated now in many centers around the world (5). Turner R. et al. analyzed EuroFlow MRD results in a real-world practice from patients with MM patients eligible for autologous stem cell transplantation (ASCT) treated with VCD as induction. Patients who achieved MRD negativity after ASCT had a significantly longer PFS than those with persisting MRD (Turner et al.). This observation is true not only in the ASCT scenario, but also for older patients who are not eligible for ASCT, and treated with more intensive combination of drugs where MRD negativity is an important parameter for prognosis (6).

Another validated method to evaluate MRD relies on Next Generation Sequencing (NGS). This method was tested not only in clinical trials but also in a real-world practice (7). Also, for innovative treatment strategies like CAR-T therapy in the MM relapse setting, MRD evaluation becomes an important prognostic factor. (8) Wong et al. performed a retrospective analysis of 54 BCMA-CAR-T treated MM patients from five different clinical trials at the University of California San Francisco. Patients achieving MRD-NGS below the detectable limit at a sensitivity of <10^−6^ had a better PFS than those with detectable disease at one month and three months after starting treatment.

Continuous MRD monitoring based on bone marrow (BM) analysis remains challenging because BM aspiration is an invasive procedure that cannot be repeated frequently. BM-based assays do not allow for the detection of extramedullary disease, which is increasingly seen in the clinic. From this moment on, MRD monitoring during treatment, and particularly after therapy, currently relies mainly on other less-invasive (but less sensitive) techniques, such as serum-based assessment in addition to imaging. Therefore, MRD assays based on peripheral blood (PB) would be worthwhile (9). Mass spectrometry techniques have recently been used to detect M-protein in serum with higher sensitivity than the current electrophoretic methods (10). Many groups have also investigated the role of circulating plasma cells (CPCs) at diagnosis. CPCs are becoming recognized as an independent prognostic factor in MM (including smoldering MM as well) both at diagnosis and after starting therapy (9, 11, 12).

Dhakl B.et al compared the performance of a non-invasive, circulating tumor DNA (ctDNA)-based MRD assay with multiparameter flow cytometry (MFC) of marrow aspirate to predict relapse in ASCT recipients with MM. MRD assessment using ctDNA was retrospectively analyzed on 80 plasma samples from 28 patients collected at different time points, post-AHCT. The median PFS for ctDNA-positive patients was 31 months, and that for the ctDNA-negative patients was 84 months (HR: 5.6; 95%CI: 1.8-17; p=0.0003) (Dhakal et al.).

MRD evaluation in MM treatment scenario is nowadays an important tool not only as prognostic marker but also for a risk based guided therapeutic strategy (13). Soon, the MM treatment decision, probably will be guided by sequencing evaluation of MRD (14).

In this special issue of Frontiers in Oncology | Hematologic Malignancies, a total of 8 papers related to MRD in MM are presented, these include: i) a retrospective validation of Euroflow MRD in Real-World MM patients (Turner et al.); ii) an assessment of MRD using circulating tumor DNA from patients post-ASCT (Dhakal et al.); iii) a single institution analysis of MRD in patients treated with BCMA CAR-T Cells (Wong et al.); iv) a retrospective analysis of MRD significance in patients who received ASCT (Sun et al.); v) a study evaluating bone marrow and apheresis samples with a highly sensitive 10-color MFC panel (Riebl et al.); vi) a review of the possible role of quantifying measurable clonal plasma cells in stem cell grafts (Seval et al.); and vii/viii) two studies about the state of the art of MRD evaluation in MM. (Charalampous and Kourelis, Alonso and Lahuerta).

## Author contributions

All authors listed have made a substantial, direct, and intellectual contribution to the work and approved it for publication.
